# Quantitative evaluation of the strategy to eliminate human African trypanosomiasis in the Democratic Republic of Congo

**DOI:** 10.1186/s13071-015-1131-8

**Published:** 2015-10-22

**Authors:** Kat S. Rock, Steve J. Torr, Crispin Lumbala, Matt J. Keeling

**Affiliations:** Life Sciences, Warwick University, Coventry, CV4 7AL, UK; WIDER, Warwick University, Coventry, CV4 7AL, UK; Liverpool School of Tropical Medicine, Liverpool, L3 5QA, UK; Programme National de Lutte contre la Trypanosomiase Humaine Africaine (PNLTHA), Kinshasa, Democratic Republic of Congo; Mathematics Institute, Warwick University, Coventry, CV4 7AL, UK

**Keywords:** Sleeping sickness, Gambian human African trypanosomiasis, Mathematical model, Basic reproductive ratio, Elimination, Democratic Republic of Congo

## Abstract

**Background:**

The virulent vector-borne disease, Gambian human African trypanosomiasis (HAT), is one of several diseases targeted for elimination by the World Health Organization. This article utilises human case data from a high-endemicity region of the Democratic Republic of Congo in conjunction with a suite of novel mechanistic mathematical models to address the effectiveness of on-going active screening and treatment programmes and compute the likely time to elimination as a public health problem (i.e. <1 case per 10,000 per year).

**Methods:**

The model variants address uncertainties surrounding transmission of HAT infection including heterogeneous risk of exposure to tsetse bites, non-participation of certain groups during active screening campaigns and potential animal reservoirs of infection.

**Results:**

Model fitting indicates that variation in human risk of tsetse bites and participation in active screening play a key role in transmission of this disease, whilst the existence of animal reservoirs remains unclear. Active screening campaigns in this region are calculated to have been effective, reducing the incidence of new human infections by 52–53 % over a 15-year period (1998–2012). However, projections of disease dynamics in this region indicate that the elimination goal may not be met until later this century (2059–2092) under the current intervention strategy.

**Conclusions:**

Improvements to active detection, such as screening those who have not previously participated and raising overall screening levels, as well as beginning widespread vector control in the area have the potential to ensure successful and timely elimination.

**Electronic supplementary material:**

The online version of this article (doi:10.1186/s13071-015-1131-8) contains supplementary material, which is available to authorized users.

## Background

The London Declaration on Neglected Tropical Diseases [1, 2] lists human African trypanosomiasis (HAT) as one of the diseases targeted for “elimination”. In the case of HAT, the elimination target is defined as “the reduction of gambiense HAT incidence to less than 1 new case per 10,000 population at risk, in at least 90 % of foci with fewer than 2000 cases reported globally” by 2020 and “to target zero incidence of the disease by 2030” [3]. HAT is caused by subspecies of *Trypanosoma brucei*, a protozoan parasite transmitted by tsetse flies (*Glossina* spp.). While the single name “sleeping sickness” is commonly used, the disease exists in two distinct forms. Most (>98 %) cases occur in West and Central Africa and are caused by *T. b. gambiense* transmitted by the riverine species of tsetse. This Gambian form of the disease is generally regarded as an anthroponosis [4], although parasites are sometimes detected in non-human hosts and could play a role in transmission [5]. The remaining 2 % of cases are caused by *T. b. rhodesiense* transmitted by savanna species of tsetse. Rhodesian HAT is a zoonosis typically associated with the wilderness areas of East and Southern Africa where tsetse vectors, wild reservoir hosts (e.g., warthog, buffalo) and occasionally cattle are abundant. The zoonotic nature of Rhodesian HAT means that complete elimination of the disease is unlikely [3]. Hence, the goal of elimination is specific to Gambian HAT.

The global number of reported cases per year is in line with the goal of elimination. WHO’s target is for a steady decline to 2,000 cases per year by 2020: the target for 2014 was 5,000 cases [6] compared to a reported number of 3,679 [7]. The last bastion of Gambian HAT is the Democratic Republic of Congo (DRC) where 87 % (3,206/3,679) of all cases reported in 2014 occurred. The results for 2014 are typical for the last 15 years (2000–2014): cases of HAT from the DRC have been between 58 % (11,481/19,963 in 2003) and 91 % (5,647/6,228 in 2013) of all cases reported globally [7]. Within DRC itself, the distribution of HAT is also highly heterogeneous with three provinces being particularly important. Of the 127,960 cases reported from DRC between 2000 and 2012, 46 % (58,585 cases) came from Bandundu province, 21 % (26,692 cases) from Kasaï (East and West) and 17 % (21,575) from Équateur [8]. Achieving the global elimination goal for Gambian HAT therefore depends on the impact of interventions in the DRC and these three provinces in particular.

Control of Gambian HAT in DRC, as elsewhere, has depended almost exclusively on active case detection and treatment [9]. In the DRC for instance, more than 24 million people were screened between 2000 and 2012 [8]. As HAT disease progresses through two distinct stages, each with different treatments (pentamindine for stage 1 and nifurtimox and eflorithine combination treatment (NECT) for stage 2), diagnosis therefore involves the use of multiple diagnostic tools. Diagnostic algorithms vary across space and time, however key steps include screening for infection usually via the card agglutination test for trypanosomes performed on whole blood (CATT-WB), visual confirmation of the parasite using microscopy, and “staging” disease via a lumbar puncture; if more than 5 white cells per *μ* l (microlitre) or trypanosomes are identified in cerebral spinal fluid then the patient has stage 2 disease [9]. In addition to these large-scale active programmes, some individuals self-present at medical centres following onset of symptoms of which the worst (i.e. neurological systems) occur in stage 2; this is referred to as “passive” detection. Historically, vector control has not played an important role because of the cost and logistical complexities of traditional methods of tsetse control [10] and the emphasis on active screening continues to date. A key issue is whether continuing the current strategy is likely to result in the elimination of Gambian HAT in the DRC. To address this question we have developed and applied a mathematical model of HAT to data from Yasa-Bonga and Mosango Districts in the Bandundu Province of DRC.

The use of models to analyse vector-borne diseases originates with the Ross-Macdonald equation for malaria [11–14] and developments of this model have formed the basis of several models of HAT (see review by [15]). Models of HAT differ from those of malaria in two important respects reflecting differences in the underlying biology of the two diseases. First, tsetse flies are susceptible to infection with *T. brucei* sspp. when they take their first blood from an infected host but are much less susceptible for subsequent blood-meals. Indeed some models assume that tsetse are susceptible at their first feed only and completely refractory thereafter [16, 17]. Some studies have shown that older flies can be infected and go on to develop mature infections but the extent of this feature in nature is uncertain [18]. There is however widespread agreement that there is a marked “teneral phenomenon” whereby susceptibility declines with the fly’s age [19]. Second, it is generally assumed that humans are the only host for *T. b. gambiense*. Again empirical data suggest that this might not always be the case: *T. b. gambiense* has been detected in wildlife [20] and livestock [21, 22] and experimental infections with *T. b. gambiense* have shown that they retain their pathogenicity to humans even after repeated passage through pigs [23–25]. The epidemiological significance of these empirical data is uncertain, but previous modelling has suggested that it is possible that such infected animals may be an important reservoir of infection [5]. Hence the teneral phenomenon and the potential for non-humans to act as hosts for trypanosomes need to be incorporated into models of HAT.

Model predictions for the impact of active screening on the incidence of HAT are dependent on key assumptions regarding the heterogeneity of the human population in relation to (i) their exposure to infection and (ii) their willingness to be screened. It is widely recognised that some groups of people are more likely to be bitten than others. For instance, it is self-evident that people with occupations that require them to spend extended periods in riverine habitats (e.g., fishermen, women carrying out domestic chores such as washing and collecting water) are more likely to be bitten than those who never enter such tsetse-infested habitats. Screening of local populations seldom exceeds 70 % despite efforts to mobilize and encourage the population to attend [26, 27] and there are strong indications that some groups (e.g. working adults) are consistently under represented [27]. While these observations suggest that there are strong heterogeneities in the human population, the extent of this is not well quantified and likely to vary between settings.

Our understanding of the biology, behaviour and ecology of some species of tsetse is relatively good [4, 28]. However, the important vector of *T. b. gambiense* in Yasa Bonga and Mosango is *G. fuscipes quanzensis* and for this species there are scant empirical data. Some aspects of the basic biology of all species of tsetse are very similar (e.g. producing a single offspring at ~9 day intervals, both sexes taking bloodmeals at ~3 day intervals) and we can be reasonably confident in applying general values derived from one closely related species (e.g. *G. f. fuscipes*) to provide parameter estimates such as the underlying daily mortality and feeding interval of *G. f. quanzensis*. On the other hand, there are some values that apply to *G. f. quanzensis* for which there is much less certainty and these need to be inferred during model fitting to data: e.g. the transmission efficiencies of trypanosomes between tsetse and humans and the ratio of vectors to humans.

Robust mathematical models, in conjunction with rigorous statistical fitting to available data are fundamental tools for modern public-health decision makers in many counties [29–32]. Until recently, this approach has had limited impact on health policy in impact in low- and middle-income countries and has been largely overlooked in the study of NTDs (although onchocerciasis [33] is a notable exception). The basic principles are to (i) develop a mechanistic mathematical framework that captures the salient aspects of known biology and epidemiology, (ii) match this model to available data, and (iii) use the model to predict forwards in time allowing multiple control options to be investigated. Here we apply these concepts to HAT in the DRC, to investigate the potential of current controls to achieve the WHO 2020 goals – elimination of HAT as a public health problem (less than one case per ten thousand per year) by 2020.

Many epidemiologists are familiar with statistical models, and the methods surrounding matching such models to data. Mechanistic mathematical models are simply another type of statistical model, but one in which our knowledge and assumptions about the underlying processes are explicitly formulated. This has two profound implications; the first is that in matching models and data the parameter values inferred generally relate to specific physical quantities such as average infectious periods or rates of transmission. The second is that when using the model to extrapolate forwards in time, it is possible to change some of these fundamental parameters and hence mimic the effects of control mechanisms.

Like any statistical fitting and extrapolation, different models may produce different results – just as fitting linear and non-linear statistical models can generate very different conclusions. Given that there is no right model that can capture every element of the transmission process, and since many of the mechanisms are unknown for HAT, it is vitally important that multiple modelling assumptions are tested against the available data.

In this article, a range of hypotheses are tested utilising data from a HAT-endemic region of the DRC in conjunction with mathematical models that can capture different assumptions about tsetse biology and behaviour, population level heterogeneities, and the potential existence of animal reservoirs. Under each scenario, disease dynamics are projected forwards to determine the likely time to elimination as a public health problem and if this 2020 goal can be met for HAT.

## Methods

### Data

Bandundu province accounted for almost half of the reported HAT cases in the DRC between 2000–2012. For this reason the region is of great interest when considering elimination goals. For the purposes of public health services, provinces of the DRC are partitioned into smaller administrative units known as “Zones de Santé” or health zones. Two health zones within Bandundu, Yasa-Bonga and Mosango, with a combined population of 291,567 [34, 35] are considered in this article. The WHO Atlas data for these health zones cover the years 2000–2012 and detail the total number of actively and passively detected cases in addition to the number of people actively screened across these areas [36, 37]. During these years, active detection and treatment formed the primary component of HAT control programmes in this area with no large-scale vector control interventions being implemented [8].

**Fig. 1:**
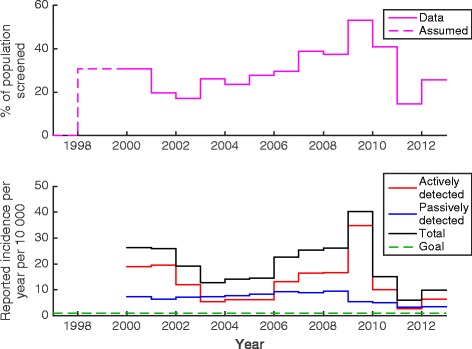
The top figure shows the total level of screening and detection in the Yasa-Bonga and Mosango health zones between 2000 and 2012. The bottom figure shows the annual incidence based on an assumed population size of 289,030 in relation to the WHO 2020 goal of elimination as a public health problem (shown in green)

During model simulation and fitting, the aggregate data from across both the health zones was used. The percentage of the population screened was taken to be the total reported number screened from the WHO Atlas relative to the estimated constant population size for the combined health zones. From this, the reported incidence per 10,000 people per year was also computed and shown in relation to the WHO 2020 goal of elimination as a public health problem (see Fig. 1).

### Model variants

**Fig. 2:**
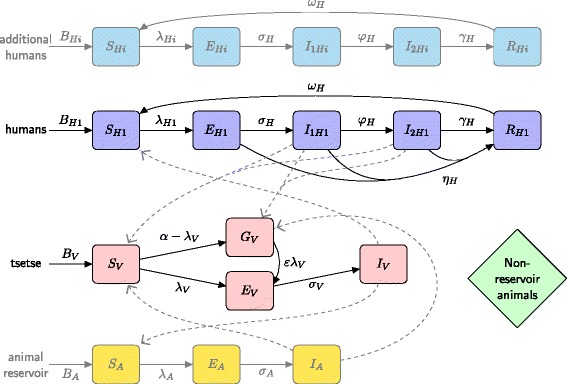
Multi-host model of HAT with various host groups able to acquire and transmit HAT infection (humans and reservoir animals), further non-reservoir animal species (others) and tsetse. Human hosts follow the progression which includes an infectious stage 1 disease, *I*_*H*1_, infectious stage 2 disease , *I*_*H*2_, and a non-infectious (due to hospitalisation) disease, *R*_*H*_. Unfed tsetse are susceptible, *S*_*V*_, and following a blood-meal become either exposed, *E*_*V*_, or have reduce susceptibility to the trypanosomes , *G*_*V*_. Tsetse select their blood-meal from one of the host species. Any blood-meals taken upon non-reservoir hosts do not result in infection. The transmission of infection between humans/tsetse and reservoirs/tsetse is shown by grey paths. Additional humans follow the same progression as the first human type but may receive more bites (high-risk) or may not participate in screening. Transmission from additional humans to tsetse is not shown here but occurs in the same way as humans to tsetse

A mechanistic, compartmental model which describes the infection dynamics in the population was developed (see Fig. 2 and full equations in SI Section 1). This model and its variants are based on a Ross-Macdonald-type formulation [11–14] with infection stages in humans, tsetse and potentially animals. In all cases the tsetse population was partitioned into four distinct classes: teneral (unfed) flies, *S*_*V*_; non-teneral (previously fed) and uninfected flies, *G*_*V*_; exposed (infected but non-infective) flies, *E*_*V*_; and infective flies, *I*_*V*_. As described previously, the teneral phenomenon means that fed tsetse are less susceptible than their teneral counterparts and so the force of infection towards them is scaled by a factor *ε* (< 1). The model incorporates natural disease history in humans following infection with individuals progressing through the classes: susceptible, *S*_*H*_; exposed, *E*_*H*_; stage 1 infection, *I*_1*H*_; stage 2 infection *I*_2*H*_; and removed (hospitalised or resting), *R*_*H*_. Here, both stage 1 and 2 of the disease were assumed to be equally infective to tsetse, whereas removed individuals were not available for blood-feeding. It was assumed that stage 2 cases either were successfully diagnosed and treated, suffered disease induced mortality (and were subsequently replaced by a new susceptible in the population) or died due to unrelated causes (natural mortality) prior to treatment; consequently all compartments only contain living individuals. It is assumed that humans received a proportion, *f*_*H*_, of all bites, with the rest taken on animals that are initially assumed not to contribute to disease transmission (non-reservoir).

There are 7 model frameworks investigated to analyse competing hypotheses:

The simple model:

1. Homogeneous human risk and behaviour model. This model used the standard assumption of solely anthroponotic disease transmission and, due to its simple formulation, provided a suitable framework in which to investigate the impact of teneral susceptibility and other fundamental parameters.

Models for heterogeneous human populations:

2. High/low-risk populations. High-risk individuals being more exposed to tsetse bites.
3. Random participation/non-participation. A fraction of the population never participates in active screening, while the remainder randomly participate based on observed screening levels.
4. High-risk and non-participation/low-risk and random participation. High-risk individuals are precisely the ones who never participate in active screening
5. All combinations of high/low risk and random/non-participation. The human population is first partitioned into low/high risk groups, and each of these is further partitioned into those that never participate in screening and those that randomly participate creating four distinct human behaviour/risk compartments.

Models for animal reservoirs:

6. Homogeneous human population and two animal populations – a reservoir (able to acquire and transmit disease) and a non-reservoir (which still receive tsetse bites).
7. The model for heterogeneous human populations, as in model 4, with additional animal reservoirs.

In all further discussion, the basic reproductive ratio, *R*_0_^2^, was computed by evaluating the square of the spectral radius of the next generation matrix (NGM) (see SI Section 2). *R*_0_^2^ was chosen as a more suitable metric over *R*_0_^2^ as it is equivalent to the average number of secondary host cases generated by a single infectious host (i.e. a full transmission-cycle) (see [15]). *R*_0_^2^ was computed in the absence of active detection and treatment, and can be viewed as a measure of transmission with only passive detection occurring. No measure was given after active screening began as the intensity of screening varied year to year and the pulsed nature of this screening does not allow for computation using the NGM approach.

### Model fitting

Large-scale active detection campaigns in the region under consideration started around two years before the data began, therefore 1998 and 1999 were taken to have the same level of screening as 2000 (see Fig. 1). Prior to 1998 it was assumed that disease was at its endemic equilibrium (including passive detection) due to the absence of widespread intervention. The compartmental ODE model used the reported percentage of active screening to simulate the effect of an annual, pulsed detection and treatment campaign between 1998 and 2012 in addition to passive detection and treatment. The sensitivity and specificity of the active diagnostic algorithm were taken to be 91 % and 99.9 % respectively based on the algorithms used by MSF in the Republic of Congo, Sudan and Uganda [38]; these algorithms involve multiple diagnostics including the Card Agglutination test for trypanosomes on whole blood (CATT-WB) and parasitological examination of cerebral spinal fluid from lumbar puncture. Upon diagnosis, true positives in the model were successfully treated (and on average were hospitalised/stayed at home for six months, as per the recommendation [26]), whereas false positives remained susceptible. Passive case detection was assumed to occur at a fixed rate with individuals self-presenting to medical facilities after developing stage 2 disease; the rate was chosen such that individuals remain in stage 2 for six months on average. Whilst there is currently no robust estimate of this period, other modelling work has estimated that the duration of stage 2 disease is nine months on average in the absence of treatment [39]. In addition, it was assumed that there was some unknown level of underreporting of cases, due to either diagnosed cases not being entered into records or death before correct diagnosis (autopsies are not routinely performed to ascertain cause of death). Estimates of underreporting for HAT are thought to be high, possibly in excess of 50 % underreporting [37, 40, 41]. The parameter, *u*, is the proportion of passive cases reported.

The model calculates the continuous disease dynamics of host and vector populations (see SI Figure S.1) and outputs the expected number of passively detected cases (all at stage 2) across each year, *P*_*M*_(*i*), and the expected number of actively detected cases (both stage 1 and 2) per annual screen, *A*_*M*_(*i*) (see SI for more details). Finally the model also computes the actual number of new cases per year; these are newly infected individuals as opposed to newly detected cases. It is noted that the number of detected cases are a product of both the natural incidence of infection and the screening effort, with these two factors difficult to untangle in reporting data.

To compare and fit the models to the number of reported (active and passive) cases, a Metropolis-Hastings MCMC algorithm was used to calculate the posterior mean and 95 % credible interval both for the unknown parameters; this allows the inference of credible intervals for the incidence of new cases each year and for the predicted years until elimination as a public health problem. The fitting procedure estimated up to 11 unknown parameters (noting that not all are needed in all cases):

1. *m*_*eff*_ = *N*_*V*_*p*_*H*_/*N*_*H*_, the effective ratio of vectors to humans if transmission probability from vector to host were one [all models]
2. *p*_*V*_, the transmission probability from host to vector [all models]
3. *ε*, the reduced susceptibility of non-teneral tsetse [all models]
4. *r*, the relative risk (via increased proportion of bites) of “high risk” individuals compared to “low risk” individuals [models 2, 4, 5, 7]
5. *k*_1_, the proportion of low-risk, participating humans [all models]
6. *k*_2_, the proportion of high-risk, participating humans [models 2, 5]
7. *k*_3_, the proportion of low-risk, non-participating humans [models 3, 5]
8. *k*_4_, the proportion of high-risk, non-participating humans [models 4, 5, 7]
9. *k*_*A*_, the ratio of reservoir animals to humans [models 6, 7]
10. *f*_*A*_, proportion of feeding on reservoir animals [models 6, 7]
11. *u*, proportion of passive cases reported [all models].

Other model parameters, for which there are estimates available, were kept fixed and are given in Table 1.

**Table 1:** Parameter notation and values used in the compartmental models

Notation	Description	Value	Source
*μ*_*H*_	Natural human mortality rate	5.4795 × 10^− 5^ *days* ^− 1^	[45]
*B*_*H*_	Human birth rate	*μ*_*H*_ *N*_*H*_	-
*σ*_*H*_	Human incubation rate	0.0833 *days* ^− 1^	[17]
*φ*_*H*_	Stage 1 to 2 progression rate	0.0019 *days* ^− 1^	[39, 46]
*γ*_*H*_	Treatment rate from stage 2	0.006 *days* ^− 1^	Assumed (see text)
	u, Proportion of passive cases reported, Varies, -		
	Frequency of screening	Annual	-
	Active screen diagnostic algorithm sensitivity	91 %	Averaged from [38]
	Active screen diagnostic algorithm sensitivity	91 %	Averaged from [38]
	Active screen diagnostic algorithm specificity	99.9 %	Averaged from [38]
	Treatment compliance	1	Assumed
*η*_*H*_	Pulsed active screening		
*ω*_*H*_	Recovery rate	0.006 *days* ^− 1^	[27]
*δ*_*H*_	Disease induced mortality	0	Assumed
*N*_*H*_	Total human population size	291567	[34, 35]
*k*_1_	Proportion of low-risk, random participation people	Varies	-
*k*_2_	Proportion of low-risk, random participation people	Varies	-
*k*_3_	Proportion of low-risk, random participation people	Varies	-
*k*_4_	Proportion of low-risk, random participation people	Varies	-
*m*	Relative tsetse density	*N*_*V*_/*N*_*H*_	-
*μ*_*V*_	Tsetse mortality rate	0.03 *days* ^− 1^	[17]
*α*	Tsetse bite rate	0.333 *days* ^− 1^	WHO 2013
*σ*_*V*_	Tsetse incubation rate	0.034 *days* ^− 1^	[47, 48]
*p*_*V*_	Probability of tsetse infection per single infective bite	Varies	-
*p*_*H*_	Probability of human infection per single infective bite	Varies	-
*m*_*eff*_	Effective tsetse density	= *mp*_*H*_	-
*ε*	Reduced non-teneral susceptibility factor	Varies	
*f*_*H*_	Proportion of blood-meals on humans	0.09	[49] (*G. fuscipes*)
*r*	Relative bites taken on “high-risk” humans compared to “low-risk”	Varies	-
*μ*_*A*_	Natural reservoir animal mortality rate	0.0014 *days* ^− 1^	Assumed
*B*_*A*_	Reservoir animal birth rate	*μ*_*A*_ *N*_*A*_	-
*σ*_*A*_	Reservoir animal incubation rate	0.0833 *days* ^− 1^	[17]
*f*_*A*_	Proportion of blood-meals on reservoir animals	Varies	-
*N*_*A*_	Reservoir animal population size	Varies	-
*p*_*A*_	Probability of reservoir animal infection per single infective bite	Varies	-

This fitting procedure maximised the binomial log-likelihood function. In essence this captures the likelihood that the observed active and passive cases each year come from the appropriate random sample of the population, where the mean is given by the deterministic dynamics (SI Section 1). Mathematically, this log-likelihood given by:

$$ LL\left(\left.\theta \right|x\right)\propto \log \left(\mathrm{\mathbb{P}}\left(\left.x\right|\theta \right)\right)={\displaystyle {\sum}_{\mathrm{i}=2000}^{2012}} ln\left[ Bin\left({A}_D(i);z(i){N}_H,\frac{A_M(i)}{N_H}\right)\right]+ ln\left[ Bin\left({P}_D(i);{N}_H,\frac{P_M(i)}{N_H}\right)\right] $$

where the model takes parameterisation *θ* = (*m*_*eff*_, *p*_*V*_, *ε*, *r*, *k*_1_, *k*_2_, *k*_3_, *k*_4_, *k*_*A*_, *f*_*A*_, *u*), *x* is the data, *Bin*(*m*; *n*, *p*) binomial probability of obtaining *m* successes out of *n* trails with probability *p. P*_*D*_(*i*) and *A*_*D*_(*i*) are the number of passive/active cases in year *i* of the data, *P*_*M*_(*i*) and *A*_*M*_(*i*) are the number of passive/active cases in year *i* of the model, and *z*(*i*) is the percentage of the population screened in year *i*.

The competing model structures were compared by performing deviance information criterion DIC [42] and computing the relative likelihood of model *i* using: exp((*DIC*_*min*_ − *DIC*_*i*_)/2) (see SI Section S4 for more details).

## Results

### Fitting models to data

**Fig. 3:**
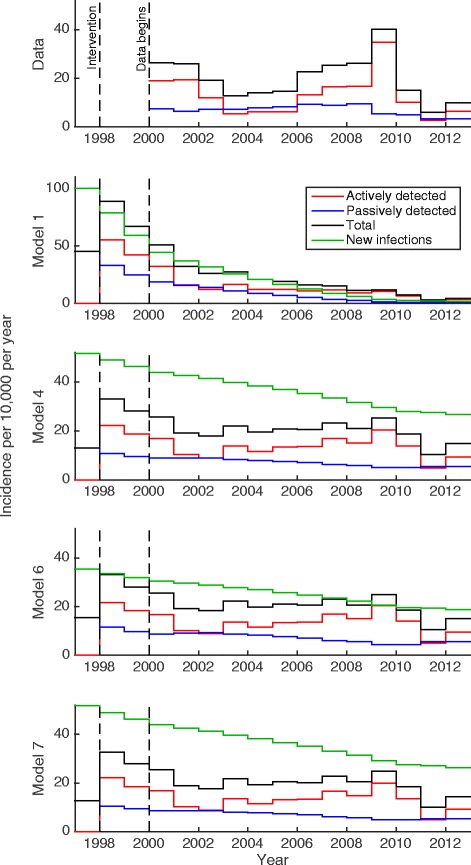
The reported incidence data and the corresponding Models 1, 4, 6 and 7 under posterior median parameterisation. Green lines show the simulated new infection incidence as well as passive, active and total reported cases under each model. N.B Model 1 has a different y-axis scale to the others figures

The three parameters *m*_*eff*_, *p*_*V*_ and *ε* affect the predicted incidence predominantly through their joint effect on the basic reproductive ratio. *R*_0_^2^ can be expressed as a function of these, for the homogeneous Model 1 taking the form:

$$ {R}_0^2=A{p}_V{m}_{eff}\left(1+B\varepsilon \right) $$

where *A* and *B* are constants given by the known parameters. The log-likelihood is not a direct function of *R*_0_^2^ but there is a very strong dependence for plausible ranges of the parameters, and the maximum log-likelihood is achieved at the same value of the basic reproductive ratio for all *m*_*eff*_ and *p*_*V*_ and *ε* (see SI Figure S.2). In further model simulation, the parameters *p*_*V*_ and *ε* were kept fixed at 0.065 and 0.05 respectively as they were non-identifiable in the model fit. During the fitting procedure, Gaussian proposals were made for *R*_0_ which were then converted to the equivalent *m*_*eff*_ value. The resulting best fit of model dynamics between 1998 and 2012 generated under the homogenous host population scenario (Model 1) are shown in Fig. 3. This simple model yielded the lowest (worst) mean log-likelihood and highest DIC of all seven models under consideration (see Table 2), indicating that heterogeneity in host population(s) is needed to capture the disease dynamics observed.

**Table 2:** Summary of models and modelling comparison results

Model	Assumptions					Relative likelihood of model (from DIC)	Notes
	Humans				Animals		
	Random participation		Non-participation				
	Low-risk	High-risk	Low-risk	High-risk			
1	✓^a^					< 10^− 100^	Least good fit
2	✓	✓				≈ 10^− 5^	Implausible parameterisation
3	✓		✓			< 10^− 100^	Poor fit
4	✓			✓		0.955	Good fit
5	✓	✓	✓	✓		0.026	No improvement over Model 4 but more parameters
6	✓^a^				✓	≈ 10^− 9^	
7	✓			✓	✓	1	Best fit

^a^Models 1 and 6 have homogeneous risk for all humans, which is equivalent to all humans being “low-risk” ✓ = included as a distinct host category in the model

All heterogeneous human models (Models 2–5) performed better than the simple model:

Model 2 (high/low risk) fitting generated very high values of *r*, the relative number of bites on high-risk individuals compared to low-risk, and very small *k*_2_, the proportion of the population in the high-risk group, indicating extreme levels of heterogeneity that are implausible. Model 3 (random/non participation) performed less well than Model 2 and only slightly better than the simple model. Model 4 (low-risk random/high-risk non-participation) was the best fitting heterogeneous human model with realistic parameterisation. It was calculated that high-risk individuals were bitten *r* = 6.6 times more and made up *k*_4_ = 7.6 % of the human population (see SI Additional file 1: Table S1). This case is also shown in Fig. 3; the generated incidence from this model is a much better fit than under the basic homogeneous assumption (Model 1). The parameters obtained for Model 5 converge to values close to those of Model 4, which is a sub-model of this more inclusive formulation. Given that Model 5 does not generate a substantial improvement in the likelihood, but requires more parameters, it has a higher DIC. This suggests that Model 4 is the most plausible human-only model that conforms with our understanding of the biology of HAT.

Again, the heterogeneity introduced by allowing for two host species (humans and reservoir animals, Model 6), significantly improved model fit over the homogeneous human model (Model 1), however Model 6 yielded a less good fit than Model 4. Results for Model 6 are shown as it forms a base-case for the inclusion of reservoir animals. Model 7 (low-risk random/high-risk non-participation and animals; i.e. Model 4 plus reservoir animals) generates a slightly improved likelihood over Model 4 when fitted to the data. The parameters of Model 7 suggest that an animal reservoir may play a small but significant role in transmission, but this may not be necessary to explain the data given that Model 4 fits almost as well.

Figure 3 shows best fits for the four models variants (Models 1, 4, 6 and 7) and the reported incidence from the WHO HAT Atlas. It is seen that Model 1 is a poor fit to the data (N.B. the y-axis range is greater than the data or other models). Model 4 is quantitatively a much better fit and generates the distinctive peak in 2009 and dip in 2011 consistent with the data. Models 6 and 7 qualitatively display the patterns seen in observed cases, however Model 7 produces a better fit than Model 6.

It is concluded that heterogeneity within the human population is needed to generate the observed cases and fitting suggests that individuals who are more exposed to tsetse bites are also not participating during active screening campaigns. Animal reservoirs alone (Model 6) seem less able to capture the disease dynamics than heterogeneous human populations (Model 4), however, this does not exclude the possibility of both heterogeneity in humans and an animal reservoir (Model 7).

Further simulations (results not shown here), demonstrated that the results for model choice are maintained for other biologically plausible choices for the parameters *f*_*H*_, the proportion of bites on humans (*f*_*H*_ = 0.4), and *μ*_*A*_, the animal death rate (*μ*_*A*_ = 0.014 *day*^− 1^). In these simulations, the relative likelihoods are similar to those shown in Table 2. Fitting yielded an altered range of possible values of *m*_*eff*_, *k*_*A*_ and *f*_*A*_, however there is currently no means of discerning the most realistic ranges without improved field or laboratory estimates for some of the uncertain parameter values.

### Predicting forwards

**Fig. 4:**
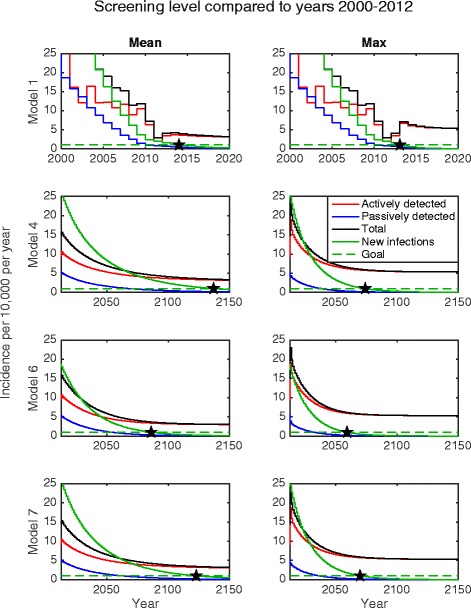
Projected incidence for HAT beginning in 2013 for Models 1, 4, 6 and 7 under posterior median parameterisation. Stars show the simulated years of elimination as a public health problem under either (i) continuing with the mean level of screening or (ii) continuing with the maximum level of screening achieved between 2000 and 2012. The imperfect specificity of the diagnostic algorithm used in active screening results in persistent reporting of actively detected cases even after elimination has been achieved

To establish the long-term impact of current active screening in the area, the model was projected forward from 2012 using either the mean or maximum level of screening observed previously (i.e. 29.9 % or 53.6 %, respectively). Under these two different scenarios the predicted time to elimination (<1 case per 10,000 people) was computed for the Models 1, 4, 6 and 7 (Fig. 4). This elimination year is defined here to be the first year in which the incidence of new infections falls below 1 in 10 000 (where the green lines cross). These predicted elimination years and the basic reproductive ratio, *R*_0_^2^, are given in Table 3 along with their 95 % credible intervals.

**Table 3:** For each biologically feasible model, this table gives the mean *R*_0_^2^ values in the absence of annual active screening as well as the predicted elimination years under either mean screening levels or maximum screening levels. 95 % credible intervals are given in brackets

Model	Pre-active screen *R*_0_^2^	Elimination as a public health problem year	
		Mean screen	Max screen
1	1.024 [1.023, 1.025]	2014 [2014, 2015]	2013 [2013,2015]
4	1.046 [1.038, 1.056]	2140 [2103, 2199]	2074 [2060, 2092]
6	1.005 [1.005, 1.005]	2077 [2072, 2101]	2059 [2050,2069]
7	1.040 [1.032, 1.048]	2124 [2098, 2176]	2072 [2059,2091]

Under the homogeneous model, the target should have already been reached as of 2014, assuming average screening levels since 2012 (Fig. 4). However, as this model variant is a poor fit for the data, this is deemed to be improbable. With heterogeneity in humans, the time to elimination becomes longer, particularly if only mean screening levels are achieved each year. In the fitted model without animal reservoirs (Model 4), even maximum screening does not achieve WHO 2020 goals, with elimination as a public health problem being reached in 2074 (95 % CI: 2060–2092). Assuming mean screening substantially delays the predicted elimination date for all models (see Table 3). Including an animal reservoir considerably increases the time to elimination with a homogeneous human population (i.e. Model 1 predicts elimination by 2013 while Model 6 predicts 2059). However the difference in elimination years for Models 4 and 7 is small.

A key observation is that the actual incidence of new infections is very different from the reported case incidence; in particular the year in which the incidence of new infections falls below 1 per 10,000 is not the same as when the reported incidence is 1 per 10,000. The passive incidence is expected to cross this threshold by as much as 74 years before the new infection incidence crosses the same threshold (Model 4, mean screening). This is due largely to high levels of underreporting of passive cases, with an estimated reporting rate of approximately 26 % (see Additional file 1: Table S2). In contrast, the long-term active case incidence is dominated by the specificity of the diagnostic algorithm and the level of active screening. Moving forwards it is crucial to define precisely what “incidence” means in terms of elimination milestones and to ensure that the number of cases continues to decline following elimination as a public health problem.

## Discussion

### Implications for 2020 goals

Model fitting indicates that the past efforts of active detection and treatment in Yasa-Bonga and Mosango health zones of the DRC have halved (52-53 % reduction) the number of new infections over a 15-year period. Although this figure is lower than the 63 % reduction in reported cases across these health zones between 2000–2012, this difference is due, in part, to the level and timing of screening which impact upon reporting in addition to the impact of underreporting. Despite this success however, these simulations suggest that we are unlikely to meet the elimination goal in these health zones by 2020 if current strategies continue unchanged; this includes sustaining the highest level (53.1 %) of yearly active screening. The results of fitting multiple model formulations to the data predicts that with the highest levels of screening the elimination target will be met between 2059 and 2092. If future screening follows the mean (29.6 %) then the predicted elimination year is increased, lying between 2098 and 2199. Whilst there has been almost 80 % reduction in reported cases for the whole DRC between 1998–2012 [7], Bandundu province of DRC remains one of the regions with the highest HAT burden globally, and it is therefore anticipated to be one of the toughest areas in which to meet this target.

This article presents a suite of models focussed on elucidating the underlying transmission pathways of *T. b. gambiense*. To compare and fit competing model structures, certain aspects which may impact transmission of this disease were not included and may account for some of the differences seen between the model output and data. We have implicitly assumed spatial homogeneity, such that all locations within the health zones experience the same level of screening and have the same underlying epidemiological risks. Spatial heterogeneity may account for the larger peak in active incidence seen in 2009 if the increase in screening was achieved through screening new areas rather than increasing the proportion screened in towns that previously participated. Throughout we have treated the data from these two health zones (which cover 6160 km^2^ [34, 35]) as a single population, whereas in reality it is comprised of multiple towns and villages. This spatial heterogeneity is computationally intensive to capture, but may have an effect on our inferred parameters and hence our long-term predictions. However, given that many regions follow the aggregate pattern, we do not expect a qualitative change to our results. Likewise, other spatial differences in the human population and tsetse habitat were not included and could affect model outcomes. It was also assumed that the passive detection rate and reporting levels were unaffected by active screening campaigns, however it may be that passive detection becomes greater with the increase in awareness of HAT in the area.

The model made use of constant human population sizes across the health zones, as very limited demographic data for these regions were available. It is believed that small variation in total population size, for example a low-level population growth rate, would not qualitatively alter the results, however longitudinal estimates of the demography of the population at a health zone level would strengthen future modelling by providing reliable values for the population sizes over time.

The model indicates that underreporting of HAT is high (possibly as much as 77 % underreporting), which is in line with values suggested in the literature [40]. Although it is not possible to determine whether this is due to under-detection of disease and a subsequent lack of identification of cause of death, or errors in data reporting. Additional data, such as testing for HAT infection posthumously, would provide future models with more robust estimates for this value.

Unfortunately there are currently no published estimates within the DRC for tsetse or animal *T. b. gambiense* infection prevalences. Whilst the model generates extremely low tsetse prevalences, in the order of 10^− 4^ to 10^− 5^, limited data for other HAT endemic countries has indicated that infection levels of T. b. gambiense in tsetse were found to be at similarly low levels, of the order 10^− 4^ [4]. New data on infection in tsetse and/or animals would enable model validation and refinement. There remain substantial uncertainties in many components of HAT transmission including relative risk of different groups of people, vector competence and the ratio of tsetse to hosts. Another modelling study which was fitted to data from Guinea [ref - 50 (Pandey)] also concluded that an animal reservoir cannot be ruled out. Gathering empirical data will be vital to improve and refine models and their predictions.

### Future for Gambian HAT

This article presents results based on defining “elimination as a public health problem” as the point where the number of new infections falls below 1 per 10,000 per year. In practice, it is hard to determine when this milestone is achieved based on active and/or passive case reporting. Passive case incidence will drop below this threshold before this target is actually met and is contingent upon the level of underreporting. The number of cases identified by active case detection will be highly dependent upon specificity and screening levels especially towards the “end-game” stages of elimination. Without introducing more specific diagnostics or diagnostic algorithms, it is possible that reported active incidence may never fall below the 1 per 10,000 threshold due to false positives. Mechanistic models can help determine the incidence of new infections and so, in the future, could provide guidance as to when controls may be lifted without recrudescence occurring.

Modelling indicates that heterogeneities in the human population play an important role in recent disease dynamics. This points towards ways of improving current active detection such as ensuring more of the population is screened, in particular those who have typically not presented at or those who are high-risk/or those who are high-risk. These results are in line with the theoretical modelling investigation by Stone and Chitnis [ref - 51] which suggests targeting high-risk individuals in screening is necessary (and may, in some cases, be sufficient) to disrupt transmission. Model results here suggest that around 8-9 % of the human population are both more exposed to the bites of tsetse and never participate in active screening; this estimate is generated by model fitting rather than social quantification. Moving forwards it would be beneficial to find suitable methods to identify such individuals so that they may be targeted during screening. Additionally, new improvements in drugs for the future treatment of HAT [9] may help improve participation in screening, for example if new drugs could be administered on an out-patient basis, or if the rest period following treatment were reduced.

These two health zones have not employed vector control as part of their intervention programme, and whilst promising new methods of tsetse control are emerging (e.g. use of tiny targets [43, 44]) their use was not modelled here. Tsetse control through tiny targets has not yet been implemented at scale in this area but has shown great potential at significantly reducing tsetse densities (up to 90 %) over a large area [43], offering a promising complementary strategy and are currently being trialled in Yasa-Bonga. In another modelling study, Pandey et al [ref - 50] find that by using a two-pronged strategy, combining active screening and vector control, the Boffa focus in Guinea is likely to meet the 2020 elimination target. In the work presented here, it is unclear from fitting to the DRC data, whether animals play an active role in transmission in this area, however tsetse control provides an intervention that circumvents heterogeneities in the human (and possible animal reservoir) population(s) by reducing biting pressure towards all hosts. Since it is a novel strategy to this area, the quantitative impact of vector control on disease cannot be directly assessed, however by utilising and fitting to data sets (both human case data and vector data) from regions with existing vector control (such as Uganda and Guinea), appropriate extensions to the existing model can be made and used to extrapolate into regions, like Yasa-Bonga and Mosango, where pre-vector control dynamics are known [27].

## Conclusions

A mechanistic modelling framework for Gambian HAT was developed to assess the success of on-going active detection and treatment in Bandundu Province of the DRC and predict the time until HAT will be eliminated as a public health problem in the region. It was concluded that active detection campaigns in this area are responsible for halving the number of new infections between 1998 and 2012, however this high-endemicity region is unlikely to achieve the WHO goal of 1 case per 10,000 people per year by 2020. The suite of models predict that this elimination target will only be reached by 2059–2092, even if the highest levels of screening achieved between 2000 and 2012 (53.1 %) continue every year. This suggests that additional strategies such as vector control and/or improved screening of the human population are required to accelerate progress against Gambian HAT.

## Additional files

Additional file 1:
**Supplementary information.** Model equations and statistical methodology. (M 19 kb) Additional file 2:
**Model code.** Matlab code for ODE model simulation.

## References

[1] Uniting to Combat Neglected Tropical Diseases. London declaration on neglected tropical diseases: Ending the neglect and reaching 2020 goals. 2012.

[2] WHO. Sustaining the drive to overcome the global impact of neglected tropical diseases. Geneva, Switzerland. 2013.

[3] Holmes P (2014). First WHO, meeting of stakeholders on elimination of gambiense Human African Trypanosomiasis. PLoS Negl Trop Dis.

[4] Welburn CS, Maudlin I (2012). Priorities for the elimination of sleeping sickness. Adv Parasitol.

[5] Funk S, Nishiura H, Heesterbeek H, Edmunds WJ, Checchi F (2013). Identifying transmission cycles at the human-animal interface: the role of animal reservoirs in maintaining gambiense human african trypanosomiasis. PLoS Comput Biol.

[6] WHO. Report of a WHO meeting on elimination of African trypanosomiasis (*Trypanosoma brucei gambiense*). 2013.

[7] WHO. Global Health Observatory Data Repository. Accessed 2015. http://apps.who.int/gho/data/node.main.A1636?lang=en.

[8] Lumbala C, Simarro PP, Cecchi G, Paone M, Franco JR, Kande Betu Ku Mesu V (2015). Human African trypanosomiasis in the Democratic Republic of the Congo: disease distribution and risk. Int J Health Geogr.

[9] WHO. Control and surveillance of human African trypanosomiasis. Geneva, Switerland: World Health Organization Techincal Report Series; 2013. Report No.: 984. 24552089

[10] Solano P, Torr SJ, Lehane MJ (2013). Is vector control needed to eliminate gambiense human African trypanosomiasis?. Front Cell Infect Microbiol.

[11] Macdonald G (1952). The analysis of the sporozoite rate. Trop Dis Bull.

[12] Macdonald G (1950). The analysis of infection rates in diseases in which superinfection occurs. Trop Dis Bull.

[13] Ross R (1911). The Prevention of Malaria.

[14] Ross R (1916). An application of the theory of probabiliteis to the study of a priori pathometry. Part I. Proc R Soc A.

[15] Rock KS, Stone CM, Hastings IM, Keeling MJ, Torr SJ, Chitnis N (2015). Mathematical models of human african trypanosomiasis epidemiology. Adv Parasitol.

[16] Hargrove JW, Ouifki R, Kajunguri D, Vale GA, Torr SJ (2012). Modeling the control of trypanosomiasis using trypanocides or insecticide-treated livestock. PLoS Negl Trop Dis.

[17] Rogers DJ (1988). A general model for the African trypanosomiases. Parasitology.

[18] Akoda K, Van den Bossche P, Marcotty T, Kubi C, Coosemans M, Dedeken R (2009). Nutritional strees affects the tsetse fly's immune gene expression. Med Vet Entomol.

[19] Haines LR (2013). Examining the tsetse teneral phenomenon and permissiveness to trypanosome infection. Front Cell Infect Microbiol.

[20] Njiokou F, Laveissiere C, Simo G, Nkinin S, Grebaut P, Cuny G (2006). Wild fauna as a probable animal reservoir for Trypanosoma brucei gambiense in Cameroon. Infect Genet Evol.

[21] Gibson WM D, Lanham SM, Godfrey DG (1978). The identification of *Trypanosoma bruceu gambiense* in Liberian pigs and dogs by isoenzymes and by resistance to human plasma. Tropenmed Parasitol.

[22] Jamonneau V, Barnabé C, Koffi M, Sané B, Cuny G, Solano P (2003). Identification of Trypanosoma brucei circulating in a sleeping sickness focus in Côte d’Ivoire: assessment of genotype selection by the isolation method. Infect Genet Evol.

[23] Van Hoff L, Henrard C, Peel E (1937). Influences modificatrices de la tranmissibilite cyclique du *Trypanosoma gambiense* par *Glossina palpalis*. Ann Soc Belg Med Trop.

[24] Van Hoff L, Henrard C, Peel E (1937). Sur le role du porc indigene comme reservoir de T*rypanosoma gambiense*. Societe Belge de Biologie.

[25] Van Hoff LMJJ (1947). Observation on trypanosomiasis in the Belgian Congo. The second Royal Society of Tropical Medicine and Hygiene Chadwick Lecture. Trans R Soc Trop Med Hyg.

[26] Mpanya A, Hendrickx D, Baloji S, Lumbala C, da Luz RI, Boelaert M (2015). From health advice to taboo: community perspectives on the treatment of sleeping sickness in the Democratic Republic of Congo, a qualitative study. PLoS Negl Trop Dis.

[27] Mpanya A, Hendrickx D, Vuna M, Kanyinda A, Lumbala C, Tshilombo V (2012). Should I get screened for sleeping sickness? A qualitative study in Kasai province, Democratic Republic of Congo. PLoS Negl Trop Dis.

[28] Torr SJ, Solano P. Olfaction in *Glossina*-host interactions: a tale of two tsetse. Olfaction in Vector-host Interactions. Wageningen Academic Publishers; 2010.

[29] Anderson RM, Grenfell BT (1985). Quantitative investigations of different vaccination policies for the control of congenital rubella syndrome (CRS) in the United Kingdom. J Hygiene.

[30] Edmunds WJ, Van de Heijden OG, Eerola M, Gay NG. Modelling rubella in Europe. Epidemiology Infection. 2000;125:617–34. 10.1017/s0950268800004660 PMC2869646 11218213

[31] Lee BY, Haidari LA, Lee MS (2013). Modelling during an emergency: the 2009 H1N1 influenza pandemic. Clin Microbiol Infect.

[32] Hollingsworth TD (2009). Controlling infectious disease outbreaks: Lessons from mathematical modelling. J Public Health Policy.

[33] UNICEF/UNDP/World Bank/WHO Special Programme for Research and Training in Tropical Diseases. Making a difference: 30 years of research and capacity building in tropical diseases. Geneva, Switzerland: WHO Library; 2007.

[34] Action Contre la Faim International. Enquête Nutritionnelle Anthropométrique: Zone de Santé de Yasa Bonga, 2009.

[35] Action Contre la Faim International. Dépistage nutritionnel rapide: Zones de santé rurale de Mosango, 2012.

[36] Simarro PP, Cecchi G, Franco JR, Paone M, Diarra A, Priotto G (2015). Monitoring the Progress towards the Elimination of Gambiense Human African Trypanosomiasis. PLoS Negl Trop Dis.

[37] Simarro PP, Cecchi G, Paone M, Franco JR, Diarra A, Ruiz JA (2010). The Atlas of human African trypanosomiasis: a contribution to global mapping of neglected tropical diseases. Int J Health Geogr.

[38] Checchi F, Chappuis F, Karunakara U, Priotto G, Chandramohan D (2011). Accuracy of five algorithms to diagnose gambiense human African trypanosomiasis. PLoS Negl Trop Dis.

[39] Checchi F, Funk S, Chandramohan D, Haydon DT, Chappuis F (2015). Updated estimate of the duration of the meningo-encephalitic stage in gambiense human African trypanosomiasis. BMC Res Notes.

[40] Mumba D, Bohorquez E, Messina J, Kande V, Taylor SM, Tshefu AK (2011). Prevalence of human African trypanosomiasis in the Democratic Republic of the Congo. PLoS Negl Trop Dis.

[41] Fevre EM, Wissmann BV, Welburn CS, Lutumba P (2008). The Burden of Human African Trypanosomiasis. PLoS Negl Trop Dis.

[42] Gelman A, Carlin JB, Stern HS, B. DD, Vehtari A, Rubin DB. Bayesian Data Analysis. Third ed. Texts in Statistical Science. London; CRC Press: 2013.

[43] Courtin F, Camara M, Rayaisse JB, Kagbadouno M, Dama E, Camara O (2015). Reducing Human-Tsetse Contact Significantly Enhances the Efficacy of Sleeping Sickness Active Screening Campaigns: A Promising Result in the Context of Elimination. PLoS Negl Trop Dis.

[44] Tirados I, Esterhuizen J, Kovacic V, Mangwiro TN, Vale GA, Hastings I (2015). Tsetse Control and Gambian Sleeping Sickness; Implications for Control Strategy. PLoS Negl Trop Dis.

[45] The World Bank. Data: Democratic Republic of Congo. Accessed 2015. http://data.worldbank.org/country/congo-democratic-republic.

[46] Checchi F, Filipe JA, Haydon DT, Chandramohan D, Chappuis F (2008). Estimates of the duration of the early and late stage of gambiense sleeping sickness. BMC Infect Dis.

[47] Davis S, Aksoy S, Galvani A (2011). A global sensitivity analysis for African sleeping sickness. Parasitology.

[48] Ravel S, Grébaut P, Cuisance D, Cuny G (2003). Monitoring the developmental status of Trypanosoma brucei gambiense in the tsetse fly by means of PCR analysis of anal and saliva drops. Acta Trop.

[49] Clausen P-H, Adeyemi I, Bauer B, Breloeer M, Salchow F, Staak C (1998). Host preferences of tsetse (Diptera: Glossindae) based on bloodmeal identifications. Med Vet Entomol.

[50] Pandey A, Atkins K, Bucheton B, Aksoy S, Galvani A, Ndeffo-Mbah M. Evaluating long term effectiveness of sleeping sickness control measures in Guinea. Parasites and Vectors, In Press. 10.1186/s13071-015-1121-x PMC4618537 26490037

[51] Stone C, Chitnis N. Implications of heterogeneous biting exposure and animal hosts on *Trypanosomiasis brucei gambiense* transmission and control. PLoS Comp Biol. 2015. 11(10): e1004514. doi:10.1371/journal.pcbi.1004514. 10.1371/journal.pcbi.1004514 PMC4591123 26426854

