# Supplementary material for: Quantitative evaluation of the strategy to eliminate human African trypanosomiasis in the Democratic Republic of Congo
Source: Parasit Vectors. 2015 Oct 22;8:532. doi: 10.1186/s13071-015-1131-8 (PMC4618948; doi:10.1186/s13071-015-1131-8)
Supplement: Additional file 1: — Supplementary information. Model equations and statistical methodology. (M 19 kb) [file 13071_2015_1131_MOESM1_ESM.pdf]

Supplementary Information
Quantitative evaluation of human African trypanosomiasis elimination strategy in the Democratic Republic of Congo

Kat S. Rock*[1,2], Steve J. Torr[2,3], Crispin Lumbala[4], Matt J. Keeling[1,2,5]

* Corresponding author: k.s.rock@warwick.ac.uk
1 Life Sciences, Warwick University, Coventry, CV4 7AL, UK
2 WIDER, Warwick University, Coventry, CV4 7AL, UK
3 Liverpool School of Tropical Medicine, Liverpool, L3 5QA, UK
4 Programme National de Lutte contre la Trypanosomiase Humaine Africaine (PNLTHA), Kinshasa, Democratic Republic of Congo
5 Mathematics Institute, Warwick University, Coventry, CV4 7AL, UK

S1 Model formulation

The HAT model equations are given below and correspond with Figure 2 (main text).

Human hosts are assumed to be in one of four distinct classes: either low-risk and randomly participant in screening (subscript $H1$), high-risk and random participants ($H2$), low-risk and never participate in screening ($H3$) or high-risk and never participate. Tsetse bites are assumed to be taken on humans or animals. The model incorporates reservoir animals which can become infected and assumes that the remainder of the bites are taken on non-reservoir animal species which do not need to be explicitly modelled.

$$
\text{Humans}\begin{cases}
\frac{\mathrm{d}S_{Hi}}{\mathrm{d}t} &= \mu_H N_{Hi} + \omega_H R_{Hi} - \alpha m_{\text{eff}} f_i \frac{S_{Hi}}{N_{Hi}} I_V - \mu_H S_{Hi}\\
\frac{\mathrm{d}E_{Hi}}{\mathrm{d}t} &= \alpha m_{\text{eff}} f_i \frac{S_{Hi}}{N_{Hi}} I_V - (\sigma_H + \mu_H) E_{Hi}\\
\frac{\mathrm{d}I_{1Hi}}{\mathrm{d}t} &= \sigma_H E_{Hi} - (\varphi_H + \mu_H) I_{1Hi}\\
\frac{\mathrm{d}I_{2Hi}}{\mathrm{d}t} &= \varphi_H I_{1Hi} - (\gamma_H + \mu_H) I_{2Hi}\\
\frac{\mathrm{d}R_{Hi}}{\mathrm{d}t} &= \gamma_H I_{2Hi} - (\omega_H + \mu_H) R_{Hi}
\end{cases}
$$

$$
\text{Animals}\begin{cases}
\frac{\mathrm{d}S_A}{\mathrm{d}t} &= \mu_A N_A - \alpha m_{\text{eff}} f_A \frac{S_A}{N_A} I_V - \mu_A S_A\\
\frac{\mathrm{d}E_A}{\mathrm{d}t} &= \alpha m_{\text{eff}} f_A \frac{S_A}{N_A} I_V - (\sigma_A + \mu_A) E_A\\
\frac{\mathrm{d}I_A}{\mathrm{d}t} &= \sigma_A E_A - \mu_A I_A
\end{cases}
$$

$$
\text{Tsetse}\begin{cases}
\frac{\mathrm{d}S_V}{\mathrm{d}t} &= \mu_V N_H - \alpha p_V \big(\sum_i f_i \frac{(I_{1Hi} + I_{2Hi})}{N_{Hi}} + f_A \frac{I_A}{N_A} \big) S_V - \mu_V S_V\\
\frac{\mathrm{d}E_{1V}}{\mathrm{d}t} &= \alpha p_V \big(\sum_i f_i \frac{(I_{1Hi} + I_{2Hi})}{N_{Hi}} + f_A \frac{I_A}{N_A} \big) (S_V + \varepsilon G_V) - (3\sigma_V + \mu_V) E_{1V}\\
\frac{\mathrm{d}E_{2V}}{\mathrm{d}t} &= 3\sigma_V E_{1V} - (3\sigma_V + \mu_V) E_{2V}\\
\frac{\mathrm{d}E_{3V}}{\mathrm{d}t} &= 3\sigma_V E_{2V} - (3\sigma_V + \mu_V) E_{3V}\\
\frac{\mathrm{d}I_V}{\mathrm{d}t} &= 3\sigma_V E_{3V} - \mu_V I_V\\
\frac{\mathrm{d}G_V}{\mathrm{d}t} &= \alpha \Big(1 - p_V \big(\sum_i f_i \frac{(I_{1Hi} + I_{2Hi})}{N_{Hi}} + f_A \frac{I_A}{N_A} \big) \Big) S_V\\
& \quad -\alpha p_V \varepsilon \big(\sum_i f_i \frac{(I_{1Hi} + I_{2Hi})}{N_{Hi}} + f_A \frac{I_A}{N_A} \big) G_V - \mu_V G_V
\end{cases}
\tag{S1.1}
$$

N.B. Here the $N_H = \sum_i N_{Hi}$ and the actual number of vectors is $S_V, E_{1V}, E_{2V}, E_{3V}$ and I_V multiplied by N_V/N_H.

$\sum_i f_i = f_H$ i.e. the total proportion of tsetse bites taken on humans. s_i is the relative availability/attractiveness of different host types, so for the 4 different humans types (low/random participant, high/random, low/non-participant, high/non), where high risk humans are r-fold more likely to receive bites, $s = (1, r, 1, r)$. The f_i's are calculated using $f_i = \frac{s_i N_{Hi}}{\sum_j s_j N_{Hj}}$.

S1.1 Basic reproductive ratio

The next generation matrix (NGM) (see [S2]) is used to compute R_0 before active case detection and treatment began (but includes passive case detection and treatment).

Transmissions:

$$
T = \left(\begin{array}{ccccccccccccc}
 & & & & & & & & & & & & \alpha p_H f_{H1} \\
 & & & & & & & & & & & & 0 \\
 & & & & & & & & & & & & 0 \\
 & & & & & & \mathbf{0} & & & & & & \alpha p_H f_{H2} \\
 & & & & & & & & & & & & 0 \\
 & & & & & & & & & & & & 0 \\
 & & & & & & & & & & & & 0 \\
 & & & & & & & & & & & & \alpha p_H f_A \\
 & & & & & & & & & & & & 0 \\
0 & A\dfrac{f_{H1}}{N_{H1}} & A\dfrac{f_{H1}}{N_{H1}} & 0 & A\dfrac{f_{H2}}{N_{H2}} & A\dfrac{f_{H2}}{N_{H2}} & 0 & A\dfrac{f_A}{N_A} & 0 & 0 & 0 & & 0 \\
 & & & & & & & & & & & & 0 \\
 & & & & & & \mathbf{0} & & & & & & 0 \\
 & & & & & & & & & & & & 0
\end{array}\right) \tag{S1.2}
$$

where $A = \alpha p_V N_V \dfrac{(\mu_V + \varepsilon\alpha)}{\alpha + \mu_V}$

Transitions:

$$
\Sigma = \begin{pmatrix} B & & \mathbf{0} & \\ & B & & \\ & & C & \\ \mathbf{0} & & & D \end{pmatrix} \tag{S1.3}
$$

where

$$
B = \begin{pmatrix} -\sigma_H - \mu_H & 0 & 0 \\ \sigma_H & \varphi_H - \mu_H & 0 \\ 0 & \varphi_H & -\gamma_H - \mu_H \end{pmatrix}
$$

$$
C = \begin{pmatrix} -\sigma_A - \mu_A & 0 \\ \sigma_A & \varphi_A - \mu_A \end{pmatrix} \tag{S1.4}
$$

$$
D = \begin{pmatrix} -3\sigma_V - \mu_V & 0 & 0 & 0 \\ 3\sigma_V & -3\sigma_V - \mu_V & 0 & 0 \\ 0 & 3\sigma_V & -3\sigma_V - \mu_V & 0 \\ 0 & 0 & 3\sigma_V & -\mu_V \end{pmatrix}
$$

The NGM, K, is given by:

$$
K = -T\Sigma^{-1} \tag{S1.5}
$$

and the R_0 is the spectral radius of K:

$$
R_0 = \rho(K) \tag{S1.6}
$$

In all further discussion, R_0^2 is used as a measure of the reproductive ratio, due to its biological representation of full cycle or host-to-vector-to-host transmission.

S2 Model output

The compartmental ODE model is simulated to compute the disease dynamics in humans, animals and tsetse (see Figure S1). The total annual passive reported cases for year, T is calculated by integrating over the new hospitalisations from self-presentation multiplied by the reporting parameter, u, to compensate for underreporting of passive cases:

$$P_M = u \sum_i \int_T^{T+1} \gamma_H I_{2Hi}(t)\,\mathrm{d}t \tag{S2.1}$$

where $i \in$ all human types), whereas the active number of reported cases is given as:

$$A_M = \sum_j \text{proportion screened} \;\times\; \text{test sensitivity} \;\times\; \text{compliance} \times (I_{1Hj}(T) + I_{2Hj}(T)) \tag{S2.2}$$

where $j \in$ random participants. The number of reported cases seen under the model is also shown in Figure S1.

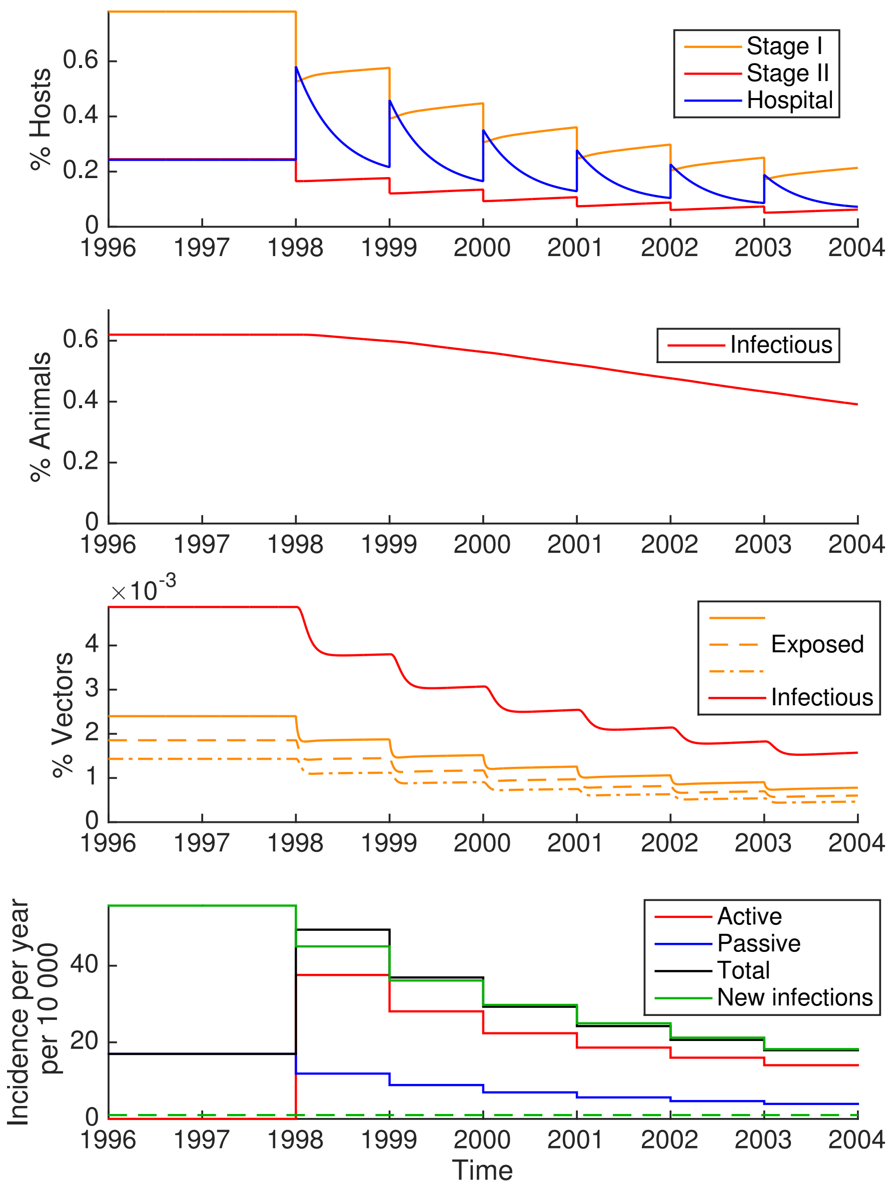

Figure S1: Example disease dynamics of the human, animal, tsetse model. The top 3 graphs show the continuous disease dynamics generated by the ODE model, with active, pulsed screening taking place annually from 1998 and a passive reporting level of $u = 0.32$. The bottom graph shows the incidence per year per 10,000 which is computed after obtaining the solutions to the ODE (see (S2.1) and (S2.2)).

S3 The homogeneous case

In the simple case with homogeneous human risk/behaviour and no animal reservoirs, the relationship between the R_0 and p_V, ε and m_{eff} is given by:

$$R_0^2 = A p_V m_{\text{eff}} (1 + B\varepsilon)$$

where $A = 0.7668$ and $B = 11.1$ for the given parameters in Table 1 with $u = 0.32$ (see main article).

Consequently, there are whole regions, rather than points in parameter space which yield the maximum likelihood due to the strong correlation of vector-related parameters. In particular, the effects of m_{eff} and p_V are indistinguishable (for the same R_0^2 it does not matter how m_{eff} and p_V are chosen, the log-likelihood will be the same), whereas ε impacts the force of infection term (a rate) and so the same R_0^2 value may give a different likelihood (see Figure S2).

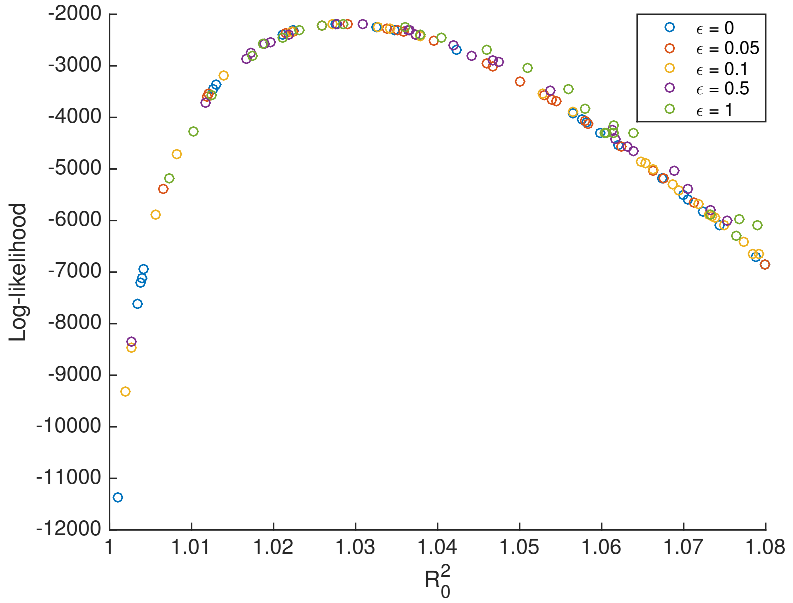

Figure S2: Scatter plot of the log-likelihood function against R_0^2 under Model 1 for fixed $u = 0.32$. Whilst the same R_0^2 generates slight variation in the value of the likelihood with varying ε, the maximum likelihood is still achieved at the same R_0^2 (≈ 1.03)

S4 Parameters and credible intervals

Imputation was performed using the standard Metropolis-Hastings MCMC algorithm with a Gaussian random walk generating sample proposals. The chain was thinned (keeping 1 out of every 100 steps) to reduce autocorrelation between samples. As there is little existing information in the literature for many of the target parameters, uniform priors were taken for all but one of the parameters (see Table S1). Posterior distributions of the fitted parameters for Models 1, 4 and 7 are shown in Figure S3. Mean acceptance rates varied between Model variants and lay between 30-75%. Table S2 gives the mean and 95% credible interval for the fitted model parameters for Models 1, 4, 6 and 7.

Model selection was performed using the popular deviance information criterion (DIC),

$$DIC = -2LL(\bar{\theta}) + 4Var(LL(\theta)) \quad \text{(S4.1)}$$

which assigns a lower score to models with high posterior mean log-likelihood whilst penalising models with a larger number of parameters [S3]. The relative likelihood of model i was computed using,

$$\text{Relative } DIC = \exp\left((DIC_{min} - DIC_i)/2\right) \quad \text{(S4.2)}$$

and was used to compare models (see Table 3 in main text). It was found that there is statistical support for both Models 4 (Relative DIC = 0.83) and 7 (Relative DIC = 1). Since DIC is known to favour over-fitted models [S1], both models were considered to be similarly supported by the data despite the marginally lower DIC score for Model 7. All other models were found to be less well supported by current data.

Parameter	Prior
R_0	U(0, ∞)
r	U(1, 100)
k_1	U(0, 1)
k_2	U(0, 1)
k_3	U(0, 1)
k_A	U(0, ∞)
f_A	U(0, 0.91)
u	Beta(2,2)

Table S1: Prior distributions for target parameters

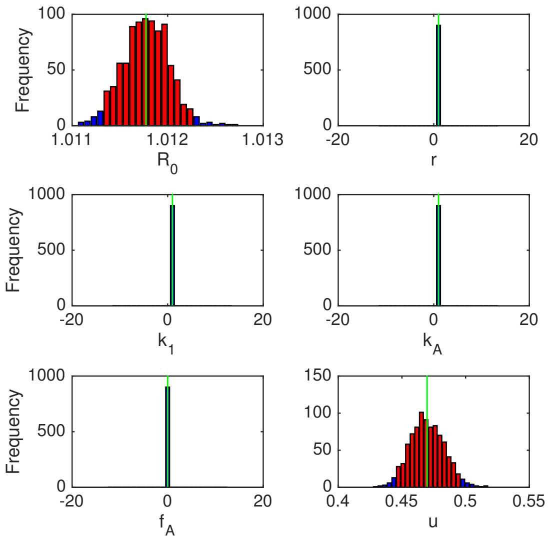

(a) Model 1

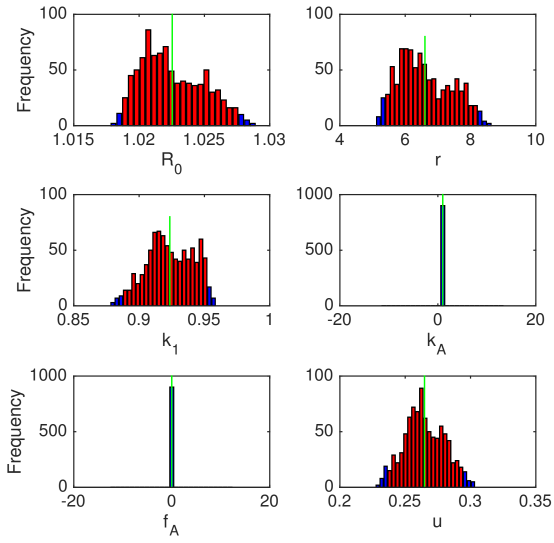

(b) Model 4

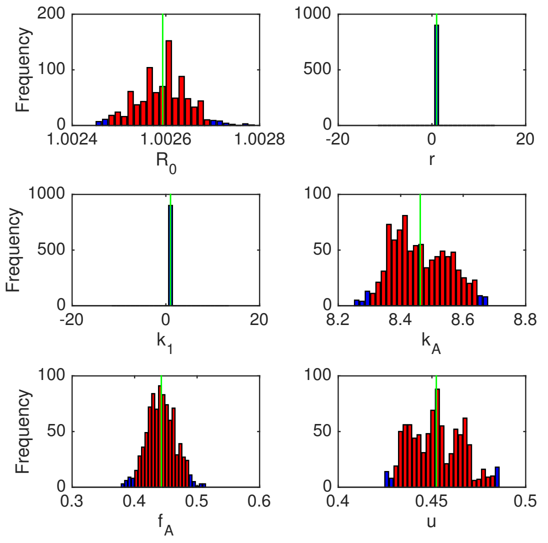

(c) Model 6

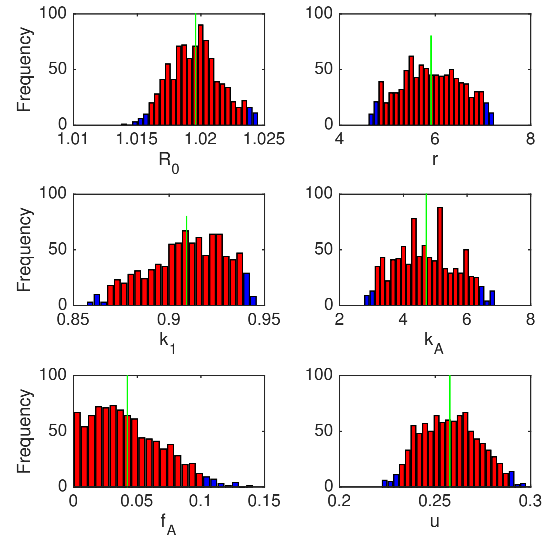

(d) Model 7

Figure S3: Posterior distributions of fitted parameters in Models 1, 4, 6 and 7

	Model 1		Model 4		Model 6		Model 7	
	Mean	95% CI	Mean	95% CI	Mean	95% CI	Mean	95% CI
R_0	1.011	[1.011, 1.012]	1.023	[1.019,1.027]	1.003	[1.002, 1.003]	1.020	[1.016,1.024]
R_0^2	1.024	[1.023, 1.025]	1.046	[1.038,1.056]	1.005	[1.005, 1.005]	1.040	[1.032,1.048]
m_{eff}	13.21	[13.20, 13.22]	6.56	[5.82, 7.29]	3.26	[2.80,3.81]	6.70	[5.78,7.63]
r	1	-	6.60	[5.35, 8.18]	1	-	5.91	[4.79, 7.07]
k_1	1	-	0.924	[0.889, 0.952]	1	-	0.909	[0.869, 0.940]
k_2	0	-	0	-	0	-	0	-
k_3	0	-	0	-	0	-	0	-
k_A	-	-	-	-	8.46	[8.30, 8.64]	4.72	[3.12,6.46]
f_A	-	-	-	-	0.443	[0.397, 0.487]	0.042	[0.002,0.103]
u	0.470	[0.445,0.497]	0.265	[0.236,0.295]	0.452	[0.430, 0.483]	0.258	[0.231,0.287]

Table S2: Parameter means and 95% credible intervals

References

[S1] T Ando. Predictive Bayesian model selection. *American Journal of Mathematical and Management Sciences*, 31:13–38, 2011.

[S2] O Diekmann, J A P Heesterbeek, and M G Roberts. The construction of next-generation matrices for compartmental epidemic models. *Journal of the Royal Society Interface*, 7:873–885, 2010.

[S3] A Gelman, J B Carlin, H S Stern, D B Dunson, A Vehtari, and D B Rubin. *Bayesian Data Analysis*. CRC Press, 2013.
